# 

**Published:** 1884-11

**Authors:** 


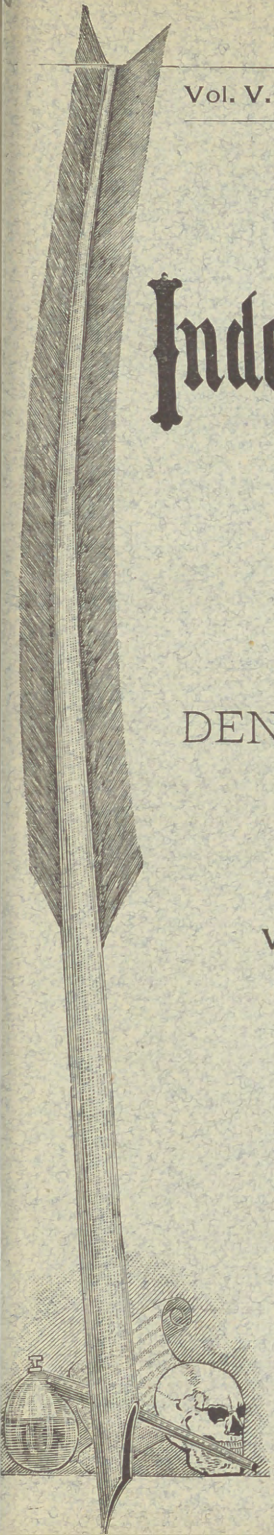

Vol. V. NOVEMBER, 1884. No. 11.

THE

### Independent Practitioner:

A MONTHLY JOURNAL

DEVOTED TO

### DENTAL AND ORAL SCIENCE.

W. C. BARRETT, M. D., D. D. S.,

EDITOR.

THE NEW YORK DENTAL JOURNAL ASSOCIATION,

PUBLISHERS,

No. 35 West 46th Street, New York.

AND

11 W. Chippewa St., Buffalo, N. Y.

$2.50 Per Annum in Advance, - - - - Single Copies, 25 Cents.

Entered at the Post-Office, Buffalo, N. Y., as Second-Class matter.

